# On opposing effects of emotion on contextual or relational memory

**DOI:** 10.3389/fpsyg.2013.00103

**Published:** 2013-03-01

**Authors:** Yi-Chieh Chiu, Florin Dolcos, Brian D. Gonsalves, Neal J. Cohen

**Affiliations:** Department of Psychology, Cognitive Neuroscience Division, University of IllinoisUrbana-Champaign, IL, USA

## Introduction

An extensive literature has firmly established the role of emotion on memory. For example, behavioral experiments have shown robustly that emotional stimuli are better remembered than neutral stimuli (see Mather and Sutherland, 2011). Mechanisms identified for such enhancements in memory include prioritized initial perceptual processing of emotional stimuli (Nummenmaa et al., 2006; Phelps et al., 2006), and amygdala (AMY)-dependent neurohormonal changes that modulate memory processes supported by the hippocampus (HC) and surrounding medial temporal lobe (MTL) structures (McGaugh, 2000), leading to memory enhancements for emotional material (Dolcos et al., 2012) as well as making such information more resistant to forgetting over time (Dolcos et al., 2005; Ritchey et al., 2008).

A caveat to the above-mentioned phenomena is that they tend to be concerned with the remembering of individual *items* in isolation, such as a word or an image. But interest in the effects of emotion on memory for real-world events, involving more than solitary items, has led to research on emotion's effects on memory for items *in the context of* or *in relation to* other items (*contextual* or *relational* information). Puzzlingly, while this recent research has confirmed the enhancing effects of emotion on memory for individual items, outcomes for the effects of emotion on memory for accompanying contextual or relational information have been contradictory, with some studies showing enhanced remembering, while others reporting impaired performances, and some reporting no effect of emotion.

In this article, we highlight two issues that that may help toward resolving the seeming contradictions in the pattern of results in this literature. The first is the need for a more nuanced conceptualization and nomenclature of the types of memory representations being tested. The second is the necessity of considering the differential engagement of HC-dependent, *relational* memory representations (Cohen and Eichenbaum, 1993; Cohen et al., 1999; Eichenbaum and Cohen, 2001), as opposed to item memory representations, that might be taxed by various experiments.

## Inconsistent findings on memory for contextual/relational information

Research on the effects of emotion on memory for contextual or relational information shows decidedly mixed results (Table 1). Some studies find *enhanced* memory for such information—e.g., better remembering of color information associated with emotional words or scenes (Doerksen and Shimamura, 2001; D'Argembeau and Van der Linden, 2005; MacKay and Ahmetzanov, 2005), screen location memory of negative arousing scenes (Mather and Nesmith, 2008), and temporal order of emotional items within a list (Schmidt et al., 2011). By contrast, other investigations find *impaired* memory for contextual or relational information—e.g., less detailed memory for scene contexts that form the background for centrally presented emotional items (Kensinger et al., 2007, experiments 1–3), and worse memory for cognitive tasks performed on items (Kensinger and Schacter, 2006b; Cook et al., 2007; but see Kensinger and Schacter, 2006a), for relations of objects superimposed on emotional scenes (Touryan et al., 2007; Rimmele et al., 2011), and for relational bindings between item pairs (Mather and Knight, 2008; Nashiro and Mather, 2011; Pierce and Kensinger, 2011; but see Guillet and Arndt, 2009). Finally, other studies do not find any differences in memory for contextual/relational information for emotional vs. neutral trials (Sharot and Phelps, 2004; Mather et al., 2009).

**Table 1:** **Sample of relevant studies on emotion's effect on contextual and relational information, organized by outcome and study design**.

**Enhanced**	**Impaired**
**Source memory** • Word-color, word-color frame (Doerksen and Shimamura, 2001) • Word-location (MacKay and Ahmetzanov, 2005) • Word-color, word-location (D'Argembeau and Van der Linden, 2004) • Picture-location ◦ (Mather and Nesmith, 2008) ◦ (Nashiro and Mather, 2011) • Scenes-location, within list order (Schmidt et al., 2011) • Temporal context: predictive preceding neutral item (Knight and Mather, 2009) • Word-task (seen vs. imagined) (Kensinger and Schacter, 2006b) **Paired Design** • Taboo word-neutral word in sentence, or taboo word-word pairs (Guillet and Arndt, 2009) **No effect of emotion** **Source Memory** • Scene-task association (color vs. visual complexity judgment) (Sharot and Yonelinas, 2008) • Paired scene-location (Mather et al., 2009) **Paired design** • Central neutral word-peripheral emotional/neutral word (Sharot and Phelps, 2004)	**Source memory** • Word, picture-task (animacy vs. commonness) (Kensinger and Schacter, 2006a) • Word-task (seen vs. heard) (Cook et al., 2007) • Scene-color frame (Rimmele et al., 2011) • Scene-locations (set of 4 scenes in 1/8 locations (Mather et al., 2006) • Face-location (Mather and Knight, 2008) **Scene context** • Emotional item embedded in scene, impaired detailed memory for scene ◦ (Kensinger et al., 2007) ◦ (Christianson et al., 1991) • Neutral peripheral object embedded in emotional scene; binding of scene-object ◦ (Touryan et al., 2007) ◦ (Rimmele et al., 2011) • Temporal context: non-predictive preceding neutral item (Knight and Mather, 2009) **Paired designs** • Word-word pairs (Pierce and Kensinger, 2011) • Sound-digit, face-hat pairs (Mather and Knight, 2008) • Picture-shape pairs (Nashiro and Mather, 2011)

## Resolution of contradictions

Studies of the effects of emotion on memory for contextual or relational information vary widely in the modality and informational structure of the contents tested. We suggest that this variation among studies critically involves different types of relational content and, consequently, differences in the memory representations they test; understanding these differences may help resolve some of the seemingly contradicting findings in the literature.

### Differentiation of information content tested

In a subset of designs commonly referred to as “source” memory studies in the literature, contextual/relational information has been operationalized across a wide range of modalities, including visual-perceptual stimulus features (color, location of item), temporal information (item order within a list), or cognitive operations performed (size, animacy judgments). What gives rise to the collective term “source” used to describe the contextual/relational information in these instances, is commonalities in the way such information is structured. That is, “source” information is always far fewer in number compared to the number of trials and can be conceptualized as grouping labels for items. For example, responses in source studies consist of a limited number of alternative choices for each modality (one of two colors or lists, or a specific quadrant on the screen) which can often be specified with a single descriptor (such as “red,” “list 2,” or “animacy”). In addition, for the purpose of making more nuanced distinctions between the types of contextual/relational information tested, it is important to note that source studies have not differentiated between memory for contextual and relational information (to be discussed below). Retrieval queries in these studies have been limited to the recall or recognition of the source information for cued items; when the source is correctly remembered/ attributed, it simultaneously implies accurate memory for the content of the source itself.

In contrast to source memory studies, there are experiments in which there is a one-to-one relationship between the contextual or relational information and trials. In other words, contextual or relational information is trial-unique and as numerous as the trials. Examples include studies that use visual images as background to items, and other studies that present two items in a pair per trial. Importantly, *two* types of information content can be distinguished and tested to demonstrate memory in these designs. The first is *contextual* information content, such as studied background scenes and objects shown with emotional items. The second is *relational binding* information between the contexts and the items, such as which scene was seen with a particular item, or which two items co-occurred during study as a pair.

When the above distinction between studies is made, a pattern does emerge from the literature in terms of the effect of emotion on contextual and relational information. That is, *enhancements* of memory due to emotion have been from two specific types of source memory studies—those that involve temporal information and visual-perceptual features. For example, emotion enhances the remembering of item order within a list (Schmidt et al., 2011), color source associated with items (Doerksen and Shimamura, 2001; D'Argembeau and Van der Linden, 2005; MacKay and Ahmetzanov, 2005), and location information (Mather and Nesmith, 2008). In contrast, *impairments* of memory due to emotion tend to involve tests for *contextual* information as well as for *relational binding* information between context and items or items pairs. That is, studies consistently demonstrate worse detailed memory for scenes accompanying emotional items (Kensinger et al., 2007, experiments 1–3), and worse recognition memory for the pairing between objects on scenes (Touryan et al., 2007; Rimmele et al., 2011) or item pairs (Mather and Knight, 2008; Pierce and Kensinger, 2011).

### Relevant theories from the emotion literature

As demonstrated above, the distinctions among source, context, and relational information afford a means of conceptualizing the research findings. The importance of these distinctions is supported by two influential views in the current emotion literature, with one being relevant to studies showing enhancements in visual-perceptual source memory, the other explaining impairments in contextual information.

First, the *object-based framework* holds that arousal enhances within-object perceptual bindings intrinsic to the items which then leads to better memory retention of such bindings (Mather, 2007). This framework readily explains emotional enhancements for source memory studies where perceptual features such as the color or location are spatially proximal or overlapping with the emotional items, and thus benefit from enhanced feature-binding through focused attention attracted by the emotional stimuli (Doerksen and Shimamura, 2001; D'Argembeau and Van der Linden, 2005; MacKay and Ahmetzanov, 2005; Mather and Nesmith, 2008). Second, another view emphasizes a *trade-off* between enhancement of perceptual details for *central* information and a lack of detailed remembering for *peripheral* elements (Christianson et al., 1991), with the central vs. peripheral distinction being defined both spatially and conceptually (see Levine and Edelstein, 2009, for review). In this way, the central-peripheral trade-off view explains impaired memory for designs that test contextual information, such as scenes that serve as background for centrally presented emotional items (Kensinger et al., 2007, experiments 1–3) or for objects that are peripheral to emotional scenes (Touryan et al., 2007), since memory for central details is enhanced at the cost of peripheral information.

### Memory representations tested

Although each of the presented theories accounts for a subset of extant studies, there are findings for which they do not have direct predictions. For example, temporal source information does not have spatial properties to which the object-based framework or the central-peripheral view can apply. This is also the case for studies with paired stimuli where two items of equivalent attentional, spatial, and conceptual status are shown in any given trial. In order to conceptualize the enhancing/impairing results in a way that generalizes across a range of studies, we propose it is necessary to consider the underlying memory representations likely to result from various experimental designs.

A well-established body of research in the memory domain informs a distinction between *item* vs. HC-dependent, *relational* memory representations that support memory for relations among multiple items, and between various items and the larger context involving temporal, spatial, and situational relations (Cohen and Eichenbaum, 1993; Cohen et al., 1999; Konkel and Cohen, 2009). Since there are at least two classes of memory representations involved, it follows that research on the effects of emotion on contextual or relational memory need to consider *if* and *how* emotion may affect these representations differently.

From the pattern of results summarized above, we are proposing that there is correspondence between the subset of source studies that shows enhanced memory by emotion to likely rely on item-only memory representations, and other contextual/relational information that shows emotion-driven impairments to involve HC-dependent relational representations. To further clarify, source information enhanced by emotion tends to involve stimuli properties that are perceptual or conceptual in nature, and can thus be “fused” or “unitized” to tax only HC-independent item-memory representations (Cohen et al., 1997; Diana et al., 2008). This is the case with color or location source information that can be associated with items via a visual “snapshot,” and temporal information for multiple items that can be conceptually organized into a single, coherent sequence (as instructed in Schmidt et al., 2011). In contrast, we note that emotion seems to impair information supported by relational memory representations, such as contextual information using complex visual scenes (Kensinger et al., 2007, experiments 1–3) and relational information using item pairs (Mather and Knight, 2008; Pierce and Kensinger, 2011), unless stimulus properties allow encoding of stimuli in a non-relational manner (possibly as internally generated, unitary mental images or concepts for word-word stimuli in Guillet and Arndt, 2009) or that encoding instructions encourage the allocation of attention to visual details of the contextual information (in Kensinger et al., 2007, experiment 4). Lastly, we would further generalize that in instances where temporal, spatial, and situational information cannot be represented in a unitized manner, there would not be an enhancement of such information by emotion.

## Conclusion

To our knowledge, the proposal to conceptualize experimental designs in terms of item vs. HC-dependent relational memory representations taxed is a novel one yet to be extensively tested in emotion research. However, the interaction between the MTL and AMY during emotional memory processing, as proposed by the modulation hypothesis inspired by animal research (McGaugh, 2000), is well-established and confirmed by research in humans (reviewed in Dolcos et al., 2012). Also, there is evidence that AMY-MTL interaction during stressful events can impair HC functioning while enhancing item processing supported by the perirhinal cortex (in Mather, 2007), thus providing plausible neural mechanisms for the differential impact of emotion on these memory representations. To conclude, we propose that in addition to extant theories that are motivated by attentional biases in perception caused by emotional stimuli, future research would benefit from differentiating between source, contextual, and relational information, as well as from considering the types of memory representations taxed in various designs, as a way to further our understanding of the effects of emotion on all types of memory phenomena.
